# On the Frontline: Exploring the Perceptions of Lahore’s Inner-city School Teachers Regarding Children Problems

**Published:** 2018-10

**Authors:** Nazish IMRAN, Atif RAHMAN, Nakhshab CHAUDHRY, Aftab ASIF

**Affiliations:** 1. Dept. of Child & Family Psychiatry, King Edward Medical University/Mayo Hospital, Lahore, Pakistan; 2. Institute of Psychology, Health & Society, University of Liverpool, Liverpool, United Kingdom; 3. Dept. of Basic Sciences, King Edward Medical University, Lahore, Pakistan; 4. Dept. of Psychiatry & Behavioral Sciences, King Edward Medical University/Mayo Hospital, Lahore, Pakistan

**Keywords:** Qualitative, Teachers, Child behavior problems, School mental health promotion, School, Children

## Abstract

**Background:**

We aimed to explore inner city school teachers’ perceptions of problems faced in schools, it causes and role of schools and teachers in promoting child social and emotional well-being, using qualitative approach.

**Methods:**

Following ethical approval and informed consent, In-depth interviews were conducted in 2017 with twenty teachers belonging to four private schools in inner city area of Provincial capital, Lahore, Pakistan. Inclusion criteria were at least 12 years of formal education and minimum of 5 years’ experience in teaching profession. Framework Analysis was used to analyze data.

**Results:**

Teachers identified learning problems, inattention, disobedience, aggression, lying & disrespect as the most commonly encountered problems of children, with very few teachers mentioning emotional difficulties. Teachers view the family, parenting practices and home environment alongside media (in particular social media) as being the main causes of child behavioral problems. With prompts, however, they did identify various school and teacher-related factors having negative impact on children. Fair conceptualization of good school and good teacher was observed. Need for teacher awareness and training for strategies promoting child emotional and social wellbeing was highlighted.

**Conclusion:**

As perceived by teachers, children studying in inner city schools have several behaviour problems. Study results and the gaps identified will help in ensuring that teachers receive training targeted towards their needs. Findings of the study also substantiate the need for targeting whole school-wide preventive approach as efforts begin to implement school mental health initiative in Pakistan.

## Introduction

School experiences are important for children emotional, psychological and social development Over the past decade, a number of school-based mental health programs have been developed worldwide with aims to promote positive mental health, identify students with mental health difficulties and provide support within school settings wherever possible (1). Teachers’ role in the school mental health initiatives needs special emphasis. In low and middle-income countries (LMIC) like Pakistan, teachers are ideally positioned to serve as the frontline defense for identification and basic intervention for children with emotional & behavioral difficulties, if provided with proper training & resources (2).

Teachers training programs in Pakistan impart limited knowledge and skills in viewing children behavior from a developmental perspective or in classroom and behaviour management. Even with the on-job experience, many teachers lack the background to interact with students in manner to facilitate early intervention and prevention. Teachers own knowledge and beliefs regarding mental health influence the way they respond to student’s mental health crisis (3). Some teachers view child psychopathology to be the cause of problem behaviours (4.5), while others perceived environment as having greater role in behavior problem than genetic factors (6). Teachers tend to deliver low praise and most of their interactions with students exhibiting behavior problems are negative. Use of Punitive strategies is also common (6–8).

Teacher training is a preventive approach to improve their knowledge, skills and competence to be able to promote positive mental health, identify, refer and support students with mental health issues and facilitate students learning. WHO, Eastern Mediterranean Region recently developed a concise and practical manual of school mental health for helping educators to better support the mental health needs of their students and to take practical steps that can be implemented at low costs in school (9). For school mental health program to be satisfactorily developed in the country, an in-depth understanding of teachers’ perceptions of child emotional and behavior problems & schools and teachers’ role in it, is essential. This area is under-researched in Pakistan.

The objectives of current study were to explore: 1) what are the inner-city school teachers’ perceptions about children problems they face in schools and 2) their understanding of possible causes of these difficulties 3) their understanding of role of schools and teachers in promoting child social and emotional well-being.

## Methods

This study was conducted in 2017 in the small private schools located in inner-city areas of Urban Lahore, Pakistan having broadly same characteristics with respect of teacher-student ratio, medium of instruction, examination system (Board of Secondary and Intermediate Education, Punjab) and fee structure between 2500-5000 PKR/month. It was embedded within the pilot phase of WHO School mental health training program.

After ethical approval from the University Review Board, Principal & teachers of four schools were invited to attend informal meeting by the principal investigator, who provided an overall view of the study.

All four principals agreed to be interviewed and also facilitated in the identification of potential volunteer teachers to be interviewed based on researcher’s requirements to ensure varying experience levels of teachers, both genders as well as representation from both junior and senior classes of the school system. For teachers, inclusion criteria were at least 12 yr of formal education (equivalent to A level) and minimum of 5 years’ experience in teaching profession. Based on established qualitative methods, we identified 20 as the target number of participants in order to reach saturation in the data (10). Each school had an average of 30 teachers (Range 25–35) and 5 participants (including principal) were interviewed from each school. Participants background details & characteristics are summarized in Table 1. All 4 principals had started their career initially as teachers and later were given administrative responsibilities. Only 8 participants (including 4 principals) had degree in education before embarking in teaching career, thus majority were learning on job.

**Table 1:** Participants Characteristics (n=20)

***Variable***	***N(%)***
**Designation:**	
Principal	4(20)
Teachers	16(80)
**Gender:**	
Female	15 (75)
Male	5 (25)
**Age in categories. (yr)**	
20–30	7(35)
31–40	5(25)
41–50	4(20)
>50	4(20)
**Educational Qualifications.**	
Masters in Education(M-Ed)	8(40)
Masters degree(But not in education)	4(20)
Bachelors degree	3(15)
Intermediate	4(20)
Others	1(5) Accounting degree ACCA
**Main responsibility**	
Administrative	4 (20)
Teaching Primary classes	6(30)
Teaching secondary classes	6(30)
Teaching both primary and secondary classes	4(20)
**Involved in teaching profession since: (categories)**	
<10 yr	10(50)
10–20 yr	6(40)
20–30 yr	4(20)

### Data Collection

Data collection was done through in-depth, individual, semi-structured interview based on topic guide developed on basis of literature review and in collaboration with senior school teachers. It was aimed to gain a thorough understanding of participants perceptions of priority problems and important mental health concepts in school settings, possible causes and factors having a negative and positive impact, in particular role of schools and teachers’ in children social and emotional well-being.

The interviews ranged from 50 min to 90 min and were conducted by the principal researcher (NI) on school premises, at times convenient for the participants. Interviews of all participants, who gave their consent, were recorded and were transcribed verbatim.

### Data Analysis

The data collection and analysis were carried out simultaneously. We used framework Analysis approach to analyses data. Data analysis employed all steps of Framework analysis approach (11). i.e. familiarization with the data, themes and subthemes identification, charting & finally, data interpretation by critically examining the summaries from all participants to understands links and associations. In order to ensure the rigor and trustworthiness of the study, member checking, transparency, & reflexive journaling were utilized.

## Results

A number of themes were identified and they have been grouped according to main aims of the study and the context of being a teacher in school. Overall, remarkable consistency in themes was seen despite differences in individual participant characteristics and schools. Details of themes and sub-themes that emerged from the data within each of these categories are available from authors on request. Summary of findings are as follows:

### Problems faced by teachers in schools

Teachers described most difficulties faced in school students in term of learning problems, and students being antisocial and/or disruptive i.e. being aggressive, disrespectful, bullying other children and not following the rules.

Many children are extremely naughty and rude…They do not listen …. move away from seat often…. Throw wrappers on floors … Use of vulgar language by children, swearing, hitting is common.

Disrespect towards teachers was mentioned by many of the participants.

Problems are more with boys. Some younger teachers who were inexperienced, refused to take senior boys classes because of teasing/ whistling etc. We requested administration to have male teachers for senior boys’ classes.

In addition, bullying behaviours were also a cause of concern for teachers.

Children used to call names (bacteria) to a special child who had physical problems and was unable to walk straight.

Teachers also felt that problems in school children are increasing due to too much adverse exposures in inappropriate ages and less guidance. Surprisingly, only 2 teachers among all respondents mentioned emotional difficulties faced by the school children and that also after prompts, highlighting that children with emotional difficulties perhaps remains unnoticed and unsupported within school settings due to silent nature of their difficulties.

### Teachers’ Perceptions of Factors having adverse impact on children social and emotional well-being

Respondents believed that a number of factors, mostly outside of school have adverse impact on child social and emotional well-being.

### Factors at home

Almost all teachers emphasized the role of home environment and parental influence as most important factor in child well-being. Parents not spending time with children at home, having poor awareness of impact of their own behavior on kids (like swearing in front of child, watching inappropriate content on TV, videos, not respecting other family members at home) were other key factors at home leading to difficulties in children. Poor parenting practices, parents being too busy to look after children due to various reasons was recurrent theme in most interviews.

Parents have stopped parenting their children, they often don’t know what is happening in their child lives.

Poor guidance by elders at home due to less interactions was also highlighted. Teachers also felt that parents own bad attitude towards teachers is responsible for disrespect shown by students towards them. Family breakdown and domestic violence was also mentioned by some teachers as significant home stressors affecting children well-being.

### Factors in school

Teachers initially did not mention school or teacher factors as having possible causative role in student problems. When prompted however, teachers saw their own actions and behavior having the most significant influence on students followed by school atmosphere and peers. For example, negative behaviors and emotions may emerge if

“teachers use bad language, humiliate students / threaten them. Some teacher thinks their job is just to teach subject and that’s all…..”

But while they admitted to teachers’ bad attitude at times, they justified it by stating that, teachers themselves feel stressed out because of pressure from school administration and parents; Poor salaries and increasing workload contribute further to decrease their motivation. One teacher summed up the feelings of many teachers- the sense that teachers feel overburdened and not appreciated for their efforts.

Teaching is most deprived profession at Government level. Teachers have to meet their own survival needs too, thus are frustrated. If a teacher is frustrated and aggressive and forgets that he is supposed to guide, support and teach problem solving to students, then obviously they are unable to fulfill their role/ responsibilities.”

Principals voiced their concerns about teachers being less responsible & trustworthy and cited rapid turnover of teachers, poor teaching practices as having negative impact on students as well as school atmosphere. School atmosphere and peer interactions were also perceived by teachers as influential in developing positive behaviors in school children.

“Government schools generally have a punitive atmosphere with corporal punishments that lead to fear and resentment”

Teachers also expressed ambiguous views about various policies by Government to stop corporal punishment.

If one plants a tree and there is untidy growth, one needs to trim it. Similarly, sometimes one need to use hitting etc… for child benefit. Now a days teachers are fearful of fines/complaints.

It was noted with concern that while majority were against physical punishment, some teachers were unaware of the extreme emotional trauma a student may suffer because of humiliation.

Inconsistent policies, very frequent changes in exam system, knee jerk reactions to events also were mentioned by teachers as reasons for teachers and student dissatisfaction and having an overall negative impact on school atmosphere.

Almost all participants agreed on the importance of games and extra-curricular activities in teaching social skills and promoting child well-being, however mentioned various reasons for there being less emphasis on it in schools. Generally, the impression was that school are focusing on Academics at the cost of other activities necessary for development of a well-balanced child personality. In some cases, schools are located in small buildings without adequate play grounds, facilities to promote games/ extra-curricular activities.

### Factors in Society

Teachers expressed concerns about children being outside their homes without supervision, impact of gangs of youth on vulnerable kids in slum areas of city, overall law and order situation in country as having very negative influence on growing children.

Some children have bad company in neighborhood with older boys, they have inappropriate information at an early age which is dangerous.

Another teacher mentioned

We as society have become more materialistic and less tolerant. Our parents used to earn less but we were happy. Now both families and kids are less contented.

### Negative Role of Media/ internet

Teachers overwhelmingly expressed their reservations on increasing media usage in society and by children in particular. Wastage of time, access to inappropriate content, lack of monitoring by government organization on program content, language as well as no limit setting by parents were considered as lading to more adverse impact of technology. They specifically were concerned about sexual themes being observed in many children programs.

We are being destroyed more by media rather than terrorism. Even cartoons are full of sexual themes. If a prep class child (5 years old) is accusing a class fellow of wrongdoing with a boy in washroom, what am I supposed to think. What does he know about boys’ girls relationship… Isn’t it our society dilemma….

Values being depicted in many western cartoons are not of our society.

### Promotion of Students social and emotional well-being

All participants agreed that a good school has one of the strongest influences on grooming of young minds intellectually as well as morally. When asked about what constitutes some good school respondents stated

“Schools’ core values matter. Discipline, extracurricular activities, good infrastructure, building, grounds, resources like labs, computers all contribute to a good school.”

A good school focus on its students’ moral values in addition to education.

“A good school ensures that teachers and parents work together for children.”

Encouraging physical activities including games was felt to be an important feature of good schools due to their advantages in children well-being and promoting social skills. Teachers emphasized that good schools do have a much bigger role towards their students than to just impart education. These include teaching them life skills and empathy and caring attitude towards others.

School routines like assembly teaches children to be responsible, follow rules, make queues etc, all important lessons in life…..

Teachers saw themselves as one of the strongest influences on students emotional and social well-being. They considered themselves as role model and thus agreed that they need to focus on their own attitude, personality, punctuality and relationship with all stakeholders (parents, students and administration) to have a positive impact on students. Being able to enter teaching profession in Pakistan without any education related degree was considered as a barrier to teachers’ competency

Teachers training is very important. At the moment teacher learns on job. Schools are teachers’ nursery basically…

Regular liaison between school and parents, collaborative work was identified as very important for student well-being.

## Discussion

This study conducted in an inner-city school setting in Lahore investigated teacher’s perceptions related to students’ mental health. Respondents in our study talked about school-related problems in relation to academic difficulties and antisocial behaviours while less emphasis was seen regarding the emotional issues. Academic problems included being irregular in school attendance, lack of interest in schoolwork, not completing homework, not memorizing lessons, always failing exams, & being more interested in playing rather than studying. This emphasis on learning issues is understandable in the local context of extreme focus on linking academic excellence with success in life and schools. Acceptance of there being intellectual disability or specific learning problems as cause of academic underachievement and need for individualized education plan & curriculum modification for students with these problems is generally not considered by most teachers. Behaviour issues e.g. disobeying, lying, wandering aimlessly, speaking rudely to others, threatening and being aggressive were also major concern to teachers but not recognized as possible mental health difficulties or having biological reasons, rather they were mostly blamed on home environment and parents’ attitudes. Problems identified in our study overlaps significantly with findings reported in terms of learning problems and aggressive behavior among young people as being major areas of concern among stakeholders (12,13).

Very few teachers mentioned any emotional difficulties as a problem suggesting that internalizing disorders despite being very common in children perhaps remains unrecognized, possibly due to the comparatively silent nature of these issues. Even where noted like possible somatic symptoms which may have been due to underlying emotional distress, they were perceived by teachers as an excuse to avoid studies by students. Somatization needs to be emphasized in any school mental health initiative and teacher training in Pakistan.

Moreover, of interest was the fact that when teachers were provided with an opportunity to talk about factors causing children problems, they initially expressed their views in a very limited manner, talking only about home environment, family dynamics, and media exposure as responsible for these issues. Although this is in agreement with other studies reporting unfavorable family environment, and lack of attention or too much attention from parents as causes of child behavior problems but teachers’ perceptions did not acknowledge impact of school, neighborhood and overall society in shaping of child behavior (12,14). Negative societal attitude and peer influence i.e. peers involved in antisocial activities are also possible causes of child behavior problems (12,15). Teachers failure to acknowledge without prompts of how strong school and teacher influence may be in student well-being highlights the need for it to be emphasized in school mental health initiative in Pakistan’s local setting. Once prompted, however, teachers did acknowledge potential role of school and teachers on influencing child behavior. Still, even with prompts, they did not appear to have concept of whole school approach rather were able to relate specifically to teachers negative behaviours like rudeness, humiliating attitudes and its impact on student learning and well-being. Literature suggests that teachers recognized themselves as one of the strongest influences on student’s behavior in addition to school climate and peers (16). There is also suggestion that teacher-student relationship shapes child behavior in school as well as home context (16). Schools context has been reported to have almost as much impact on students’ emotional health as the family context (17).

Teachers in our study reported having difficulties managing children’s behavioral problems. In addition, behaviour management strategies used by teachers also appeared to be implemented in reactionary fashion following individual incidents. These findings support previous research that teachers do not consider behavior management in the whole school context despite there being well supported evidence for its positive influence (18,19). Some teachers despite alluding to the fact that students learn from their behavior admitted to relying on punitive approach to discipline kids in school. They also showed ignorance about the impact of using punitive strategies to manage behavior on child emotional well-being. We did not find any evidence form teachers of any of the schools that preventive strategies like positive atmosphere with clear rules and expectations, consistent enforcement of rules and regulations and avoiding triggers for negative behaviour were being employed. This finding that teachers are unaware of school climate influence and behaviour modification principles are important as they need to be included in any teacher training initiative.

Most of teachers had limited training in child development or behavior management principles, their perceptions may have been developed by learning on job as well as observing other teachers. Teachers are also susceptible to misperceptions because of their own background and beliefs (20). Respondents, when asked directly, were able to come up with examples of what they considered a good school and teacher but agreed that they see very few teachers around them who would fulfill their own idea of a good teacher. Except for one teacher, teaching profession was not the first career choice for any of the study respondents which are likely to affect their dedication, motivation, and effort towards their profession. In addition, poor salaries, overwork, hectic schedule and lack of incentives contribute towards teachers feeling less than satisfied with their role in schools and society. In order for any teacher training initiative to be successful, teachers not only need to be acknowledged for their important role in building society but their own stress/ concerns/ reservations should also be addressed during the program (Table 2).

**Table 2:** Implications for intervention development on basis of themes emerging from the Interviews

***Areas***	***Implications for intervention development***
Student Problems faced by teachers in school.	Impact of child emotional and behavioral issues on academic performance can be used as a powerful tool to frame intervention.
	Emphasis in intervention on silent nature of emotional difficulties and physical symptoms as common presentation of underlying emotional problems.
Causes of Student Problems	Need for session with parents to be incorporated in training manual.
	Material to be developed to be distributed to parents increasing awareness of their role in child emotional and social well-being.
	Use school mental health programs to promote
	Awareness/ emphasizes teachers’ role in positive mental health promotion.
	Teachers are stressed and overworked and stress management needs to be incorporated in intervention.
	Behaviour management principles to be emphasized as alternative to punitive discipline measures.
	Build community and family support networks and promote self-help groups.
	Guidance by teachers/ parents regarding media/ safe social media/ internet use.

The findings of this research are potentially limited by several factors. This study was conducted in an inner-city area in Lahore and by selecting respondents purposively; therefore, the results may not be generalizable to rural areas. In addition, interviewing parents and students, themselves would also have been helpful; however, time and resource limitations prevented this.

## Conclusion

Children studying in inner city schools have several behaviour problems. Teachers view the family, parenting practices and home environment alongside media (in particular social media) as being the main causes of child behavioral problems. With prompts, however, they did identify various school and teacher-related factors also have negative impact on children. Findings of study contribute to our understanding of issues faced by teachers in school and point towards areas which need to be emphasized in any school mental health initiative. It also substantiates the need for targeting whole school-wide preventive approach as efforts begin to implement school mental health initiative (Table 2). Efforts need to be directed to shift teachers’ perceptions to include their own and school’s role in promoting positive social and emotional well-being among students.

Further investigation into how these perceptions can be addressed in teacher training program is recommended.

## Ethical considerations

Ethical issues (Including plagiarism, informed consent, misconduct, data fabrication and/or falsification, double publication and/or submission, redundancy, etc.) have been completely observed by the authors.
